# Non-Invasive Radiofrequency Therapy for Musculoskeletal, Neurological, and Vascular Conditions of the Lower Limb: A Systematic Review and Meta-Analysis

**DOI:** 10.3390/jcm15062428

**Published:** 2026-03-22

**Authors:** Maria Jesus Vinolo-Gil, María José Estebanez-Pérez, Francisco Jose Vera-Serrano, Jorge Góngora-Rodríguez, Carlos Manuel Perez-Perez, Francisco Javier Martin-Vega, Ismael García-Campanario

**Affiliations:** 1 Department of Nursing and Physiotherapy, University of Cadiz, 11009 Cadiz, Spain; kiko.fisio88@gmail.com (F.J.V.-S.); jorge.gongora@uca.es (J.G.-R.); javier.martin@uca.es (F.J.M.-V.); 2 Research Unit, Biomedical Research and Innovation Institute of Cadiz (INiBICA), Puerta del Mar University Hospital, 11009 Cadiz, Spain; 3 Rehabilitation Clinical Management Unit, Interlevels-Intercenters Hospital Puerta del Mar, Hospital Puerto Real, Cadiz Bay-La Janda Health District, 11006 Cadiz, Spain; 4 Department of Physiotherapy, Faculty of Health Science, University of Malaga, 29071 Malaga, Spain; mjestebanez@ugr.es; 5 PAIDI Research Group CTS1152, Department of Medicine and Surgery, Faculty of Medicine, University of Cadiz, 11003 Cadiz, Spain; carlos.perez@uca.es; 6 PAIDI Research Group CTS391, Department of Medicine, Faculty of Medicine, University of Cadiz, 11003 Cadiz, Spain; ismael.garcia@uca.es

**Keywords:** radiofrequency therapy, diathermy, non-invasive radiofrequency, physiotherapy, lower extremity, pain, function, systematic review

## Abstract

**Background/Objectives**: Non-invasive radiofrequency (NIRF) therapy is increasingly used in physical rehabilitation. However, its efficacy across different lower limb pathologies remains unclear. This study aimed to evaluate the effects of NIRF on pain intensity and functional status in patients with musculoskeletal, neurological, and vascular conditions of the lower limb. **Methods**: A systematic review with meta-analysis of randomized controlled trials (RCTs) was conducted following PRISMA guidelines. The PubMed, Scopus, Web of Science, PEDro, and Cochrane Library databases were searched for RCTs comparing NIRF with sham, standard care, or other physical modalities. Methodological quality was assessed using the PEDro scale. Statistical analysis was performed using RevMan 5.4 to calculate Mean Differences (MD) and Standardized Mean Differences (SMD). **Results**: Nineteen RCTs comprising 911 participants were included in the qualitative review, of which 14 were included in the quantitative meta-analysis. The mean methodological quality was 7.78/10. The meta-analysis revealed favorable results for NIRF in reducing pain intensity compared to control groups (MD = −2.04; 95% CI = −3.14 to −0.93; *p* = 0.0003; I^2^ = 96%). Functional outcomes also showed significant improvement in favor of the experimental group (SMD = −0.51; 95% CI: −0.85 to −0.16; *p* = 0.004; I^2^ = 78%). Additionally, narrative synthesis indicated benefits for spasticity management (stroke) and limb volume reduction (lipedema/lymphedema). **Conclusions**: The results suggest a trend favoring NIRF for reducing pain and improving function in lower limb musculoskeletal conditions, particularly when used as an adjunct to active therapy. Evidence also suggests preliminary beneficial effects for neurological and vascular disorders. However, these findings must be interpreted with caution due to the high statistical heterogeneity observed, the broad diversity of the clinical populations included, and the wide variability in the treatment protocols applied. Further rigorous research with standardized protocols is highly recommended.

## 1. Introduction

Lower limb pathologies represent one of the leading causes of functional disability and chronic pain worldwide, generating a significant impact on patients’ quality of life and a high economic burden for healthcare systems [1]. These conditions encompass a broad etiological spectrum including musculoskeletal disorders (e.g. tendinopathies or osteoarthritis), neurological alterations (e.g. post-stroke spasticity), and vascular dysfunctions (e.g. lymphedema) [2]. Despite their diverse etiologies, these conditions converge on shared pathophysiological mechanisms at the tissue level: impaired microcirculation, chronic inflammation, and altered neuromuscular excitability. Managing these complex manifestations requires effective and multimodal rehabilitation strategies [3].

In the field of physical therapy, non-invasive radiofrequency therapy (NIRF), frequently referred to as Capacitive and Resistive Electric Transfer (TECAR) therapy or radiofrequency diathermy, has gained prominence as a modality capable of targeting these underlying physiological dysfunctions [4]. NIRF operates through the application of high-frequency electromagnetic currents (typically between 300 kHz and 1 MHz). The biological plausibility of NIRF rests on two distinct, evidence-based mechanisms of action [4,5].

Thermally, tissue resistance to the current generates deep, localized heat, leading to significant vasodilation, increased local blood perfusion, and elevated nerve conduction thresholds. This thermal pathway is clinically associated with reduced muscle spasms, enhanced tissue oxygenation, and mechanical pain relief [6]. Non-thermally (sub-thermal), the specific frequency of the current modulates cell membrane permeability and transmembrane ion channel activity. Current clinical evidence suggests this sub-thermal mechanism accelerates the clearance of inflammatory exudates and promotes lymphatic drainage without elevating tissue temperature, offering a physiological rationale for treating acute inflammatory states and edematous disorders [4,5].

Because NIRF targets this fundamental triad of microcirculatory, inflammatory, and neuromuscular dysfunctions, its clinical application has naturally expanded beyond traditional orthopedic rehabilitation. Its efficacy in reducing musculoskeletal pain and improving joint function is well-supported by recent meta-analyses [7,8] and systematic reviews [9,10]. However, a separate review point on TECAR Therapy [11] reports some contradictory results; these discrepancies often stem from a lack of standardized dosimetry, specifically the use of thermal versus sub-thermal protocols, and the high variability in control groups, which frequently range from passive placebos to active standard-of-care treatments. Furthermore, the physiological mechanisms described above conceptually justify its use in other systems. For instance, the thermal modulation of nerve conduction velocity provides a biological rationale for managing hypertonia and spasticity in neurorehabilitation (e.g., chronic stroke) [12].

Simultaneously, the non-thermal enhancement of lymphatic and venous return supports its emerging use in dermatofunctional and vascular disorders [5,13].

Despite this strong physiological rationale, a significant research gap remains. The current literature is highly fragmented, with most systematic reviews focusing exclusively on the musculoskeletal domain [7,10,14,15], overlooking the therapy’s potential in other physiological systems. It remains unclear whether the physiological benefits observed in isolated trials translate to consistent clinical outcomes across different etiological domains of the lower limb.

Therefore, to address this limitation, the aim of this systematic review and meta-analysis was to evaluate and quantify the effects of NIRF on pain intensity and functional status in patients with lower limb musculoskeletal conditions, while simultaneously critically synthesizing the available evidence regarding its impact on the neurological and vascular disorders of the lower limb.

## 2. Materials and Methods

### 2.1. Protocol and Registration

This systematic review was conducted in accordance with the PRISMA (Preferred Reporting Items for Systematic Reviews and Meta-Analyses) guidelines [16] (see Supplementary S1). The review protocol was prospectively registered in the PROSPERO database under the registration number CRD420251150173.

### 2.2. Search Strategy

A comprehensive literature search was conducted between September and November 2025 across multiple electronic databases, including Web of Science (WOS), PubMed, PEDro, Scopus, Cochrane Central Register of Controlled Trials (CENTRAL), and SciELO. The search covered all available records from database inception up to the final update on 31 October 2025. To ensure a comprehensive retrieval of relevant studies, the search strategy employed a combination of Medical Subject Headings (MeSH) and free-text keywords using Boolean operators. The strategy was structured around three main concepts: the intervention (e.g., “Radiofrequency Therapy,” “TECAR,” “Capacitive Resistive Electric Transfer,” “Diathermy”), the anatomical location (e.g., “Lower Extremity,” “Hip,” “Knee,” “Ankle,” “Foot”), and the clinical context (e.g., “Musculoskeletal,” “Pain,” “Rehabilitation,” “Physiotherapy”). Additionally, specific exclusion criteria were applied to remove studies related to invasive procedures or oncological conditions (e.g., “Ablation,” “Surgery,” “Tumor,” “Cancer”). Filters were applied to select studies involving human adults (>18 years), with no restrictions on language or publication date. To uphold transparency and reproducibility, the full search strings for all databases are provided in the Supplementary Materials. In addition to database searches, backward and forward citation searching (“snowballing”) and a screening of clinical trial registers were performed to identify grey literature.

### 2.3. Eligibility Criteria

Studies were selected based on the PICOS framework (Population, Intervention, Comparison, Outcomes, and Study Design) [17]: Population: Adults (≥18 years) with any lower limb condition, including musculoskeletal disorders (e.g., osteoarthritis, patellofemoral pain syndrome, tendinopathies, muscle injuries), post-surgical recovery, neurological impairments (e.g., diabetic neuropathy), or vascular/lymphatic conditions (e.g., lymphedema). Intervention: Non-invasive Radiofrequency Therapy (RFT). This specifically includes Capacitive and Resistive Electric Transfer (CR-RET/TECAR) and Dielectric Monopolar Radiofrequency (MDR). Although nomenclatures vary (e.g., TECAR, CRET, MDR), included devices share the same biophysical principle of delivering high-frequency electromagnetic energy (typically 448 kHz to 4.4 MHz) to biological tissues. Studies utilizing both continuous (thermal/diathermy) and pulsed (non-thermal/neuromodulatory) emission modes were included, provided the application was non-invasive.

Comparison: Sham (placebo) intervention, standard care (e.g., exercise alone, education), or other physical therapies (e.g., ultrasound, as seen in comparative trials). Outcomes: Primary outcomes included pain intensity (e.g., VAS, NPRS) and functional status (e.g., WOMAC, LEFS). Secondary outcomes included range of motion (ROM), muscle strength, and quality of life. To evaluate the clinical relevance of these outcomes, a clinically meaningful change was defined as a minimum reduction of 2 points (or 30%) on the VAS for pain, or the achievement of the established Minimal Clinically Important Difference (MCID) for functional scales. Study Design: Randomized Controlled Trials (RCTs). Regarding the timeframe, studies were included if they reported outcomes measured at least immediately post-intervention, with no further restrictions on follow-up duration.

Conversely, studies were excluded if they involved invasive radiofrequency procedures, such as nerve ablation or percutaneous neurotomy, to ensure the focus remained strictly on non-invasive rehabilitation techniques.

Furthermore, studies were ineligible if the intervention combined radiofrequency therapy with other active modalities (e.g., injections, laser, shockwave) unless the co-intervention was applied identically in the control group, as this would prevent the isolation of the specific effect of the radiofrequency.

Finally, to ensure the specificity of the electromagnetic spectrum studied, trials utilizing other thermal modalities, such as microwave diathermy (433–2450 MHz), ultrasound, or laser therapy, were not considered.

### 2.4. Study Selection and Data Extraction

The selection process was managed using the Rayyan QCRI (Qatar Computing Research Institute) platform [18]. Duplicate records were identified and removed prior to the screening process. Subsequently, two independent reviewers (M.J.V.G. and F.J.V.S.) screened titles and abstracts against the eligibility criteria. Full-text articles of potentially relevant studies were retrieved and assessed. Any discrepancies between the reviewers were resolved through discussion or, if necessary, consultation with a third reviewer (I.G.C.). Inter-reviewer agreement was assessed using Cohen’s Kappa coefficient, yielding a value of k = 0.86, indicating substantial agreement. Data were extracted using a standardized form that included the following information: study identification (author and year), sample characteristics (sample size, age, and pathology), intervention protocols (frequency, mode, dosing, and duration), details of the comparator group, and outcome results (mean and standard deviation).

### 2.5. Methodological Quality and Risk of Bias

The methodological quality of the included RCTs was assessed using the PEDro scale [19]. Studies with a score of 6 or higher were considered to have “good” to “high” methodological quality.

For the assessment of bias, the revised Cochrane risk-of-bias tool (RoB 2) [20] was employed. This tool evaluates five domains: (1) randomization process; (2) deviations from intended interventions; (3) missing outcome data; (4) measurement of the outcome; and (5) selection of the reported result. Each domain was categorized as “low risk”, “high risk”, or “some concerns”. Two independent reviewers (M.J.V.G. and F.J.V.S.) performed this assessment, and any disagreements were resolved through discussion or consultation with a third reviewer (I.G.C.). The overall risk of bias for each study and the summary across all studies were visualized using a Risk of Bias Graph and a Risk of Bias Summary. The parallel use of these tools is methodologically justified as they provide complementary perspectives: the PEDro scale evaluates the quality of the trial’s reporting and conduct (e.g., blinding, concealment), while RoB 2 focuses specifically on the internal validity and potential bias affecting the effect size estimates.

### 2.6. Statistical Analysis

Quantitative synthesis (meta-analysis) was conducted using the Review Manager software (RevMan version 5.4). For continuous outcomes, the magnitude of the effect was calculated using the Mean Difference (MD) or Standardized Mean Difference (SMD), with 95% confidence intervals (95% CIs).

Heterogeneity was assessed using the Chi-square test and the I^2^ statistic. Given the substantial heterogeneity observed, a random-effects model (Inverse Variance) was applied for all analyses. To explore the robustness of the results and the influence of individual studies on the high heterogeneity, sensitivity analyses were performed using the ‘leave-one-out’ method where data permitted. For outcomes where meta-analysis was not feasible due to insufficient data or high clinical diversity (e.g., spasticity and limb volume), a narrative synthesis was conducted. Meta-regression was considered but ultimately not performed because fewer than 10 studies were available per covariate, as per Cochrane Handbook recommendations [21].

Subgroup analyses were performed based on the etiology of the disorder (Musculoskeletal vs. Vascular/Lymphatic). The neurological group was excluded from this quantitative subgroup analysis due to the high heterogeneity of interventions and the insufficient number of trials to perform a valid statistical pooling. To manage studies with a three-arm design (e.g., experimental, sham, and control), the comparison most relevant to the study’s primary objective was selected. Specifically, comparisons against “Standard Care” were prioritized over placebo to provide a more pragmatic estimate of the therapy’s added clinical value in real-world settings. Finally, publication bias was assessed through visual inspection of funnel plots and statistically validated using Egger’s linear regression test for the primary outcomes (pain and function), as these meta-analyses included more than 10 studies [21].

## 3. Results

### 3.1. Study Selection and Characteristics

**Figure 1:**
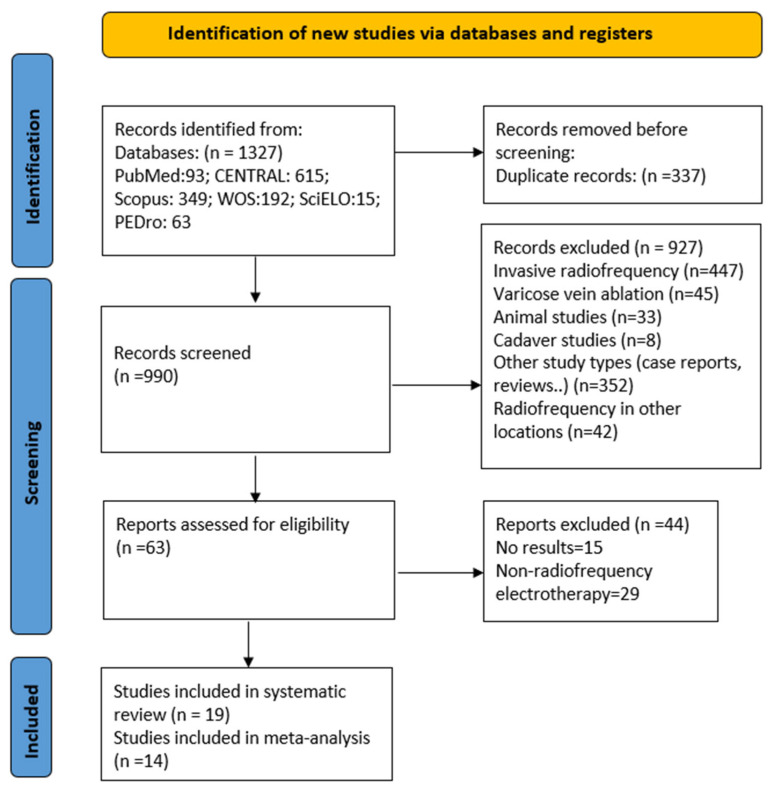
PRISMA flow diagram of the study selection process.

The study selection process is illustrated in Figure 1. Nineteen randomized controlled trials (RCTs) meeting the inclusion criteria were selected for this review.

The total pooled sample size consisted of 911 participants, with individual study sample sizes ranging from 22 participants [22] to 105 [23]. One study did not report the number of participants [24]. Geographically and temporally, the studies were predominantly conducted in Europe and Asia, with a notable increase in scientific production observed in the last five years (2020–2025). The highest number of publications originated from Spain (*n* = 6) and Iran (*n* = 5), followed by contributions from Turkey, Italy, Greece, South Korea, the United Kingdom, and Poland.

With respect to participant demographics, ages predominantly ranged from 40 to 75 years, although studies focusing on acute sports injuries or lymphatic disorders included broader age ranges. Most studies included both male and female participants; however, due to the nature of certain conditions, some trials targeted sex-specific populations: Karas, L. [24] recruited exclusively male athletes for groin pain, while studies addressing lipedema and lymphedema [25,26] recruited exclusively female participants. Notably, demographic reporting was incomplete in some trials: Davari et al. [27] and Karas et al. [24] did not explicitly report age or sex distribution.

To facilitate analysis, the studies are presented in Table 1, grouped into three main clinical domains:

- Musculoskeletal Disorders: Subdivided into acute sports injuries, knee pathology (OA and PFPS), and post-surgical interventions.
- Neurological Disorders: Categorized into chronic stroke (spasticity) and diabetic peripheral neuropathy.
- Vascular and Lymphatic Disorders: Including lymphedema and lipedema.

Within each subsection, studies are listed in reverse chronological order.

### 3.2. Methodological Quality Assessment

All included studies demonstrated good to excellent methodological quality, with scores ranging from 6 to 9. Five articles [12,22,24,36,37] were of excellent quality (9/10). The mean methodological quality of the clinical trials as measured by the PEDro scale was 7.78. Table 2 shows the detailed score for each study.

### 3.3. Risk of Bias Assessment

**Figure 2:**
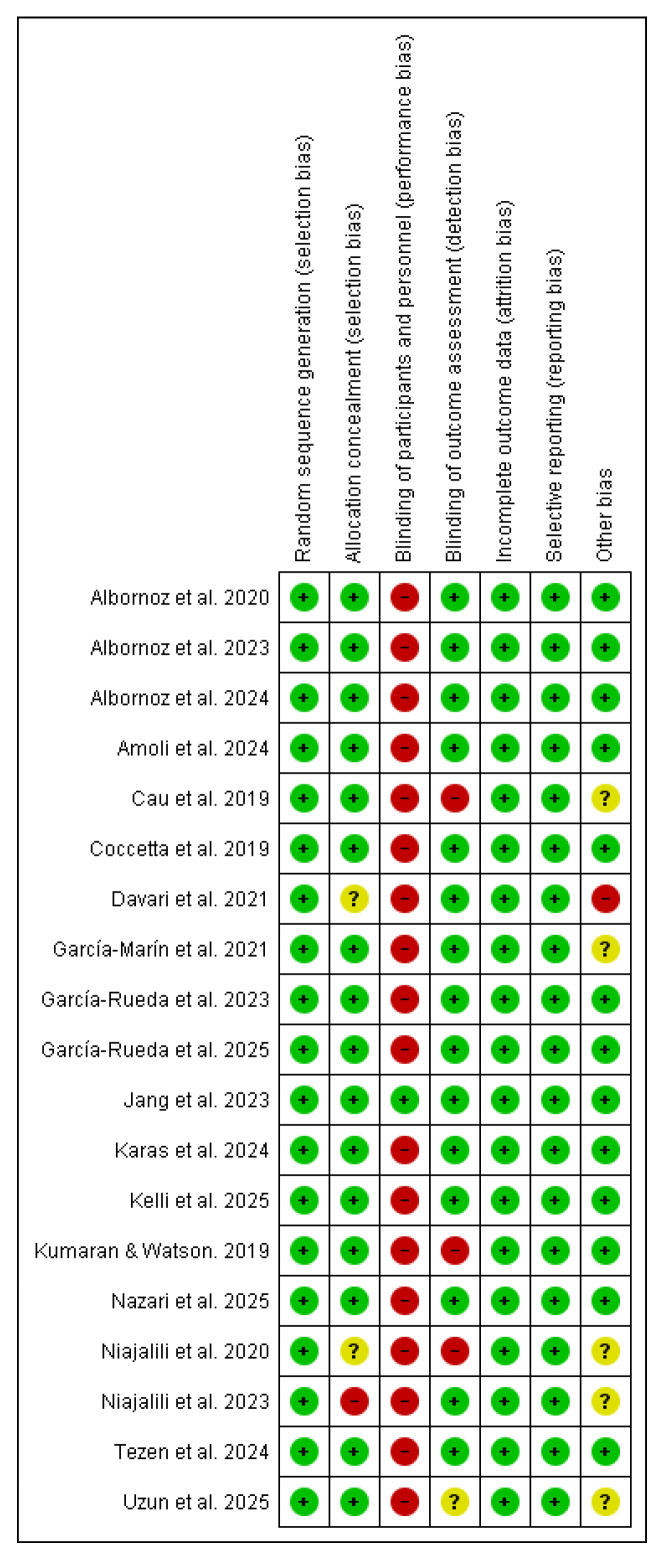
Risk of bias summary (green for low risk, yellow for unclear risk, and red for high risk).

**Figure 3:**
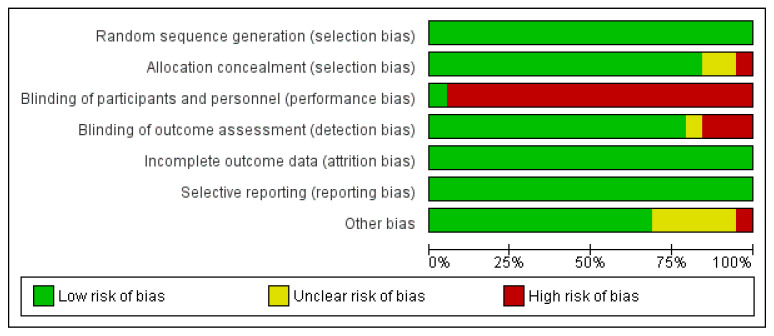
Risk of bias graph.

Regarding the risk of bias, most studies demonstrated robust methodological quality, particularly in selection, attrition, and reporting domains. The most pervasive issue was performance bias, categorized as high risk in nearly all trials due to the inherent difficulty of blinding physical interventions. Conversely, blinding of outcome assessment was generally preserved, with only three studies presenting a high risk [26,34,39]. For a detailed overview, see Figure 2 and Figure 3.

### 3.4. Narrative Synthesis of Findings

#### 3.4.1. Intervention Characteristics 

NIRF constituted the primary intervention across all experimental groups, with emission frequencies predominantly ranging between 448 kHz and 1 MHz. This spectrum encompasses the standard capacitive-resistive devices utilized in most of the included trials [12,22,26,28,29,31,32,33,34,36,37,38,39]. An exception to this range was observed in the study by Jang et al. [23], which employed a device operating at a higher frequency of 4.4 MHz. Regarding application modes, the interventions consistently combined capacitive electrodes, targeting soft and vascularized tissues, and resistive electrodes for higher-impedance structures, to ensure a comprehensive treatment of the affected area. The specific technical parameters, including frequencies, modes, and dosages for each study, are synthesized for comparative purposes in Table 3.

Crucially, the thermal dosage was strictly adjusted according to the pathology’s acuity and the therapeutic objectives. Athermal or low-intensity protocols (<10–30% output or pulsed emission) were implemented in studies addressing acute sports injuries, post-surgical recovery, and diabetic neuropathy to avoid exacerbating inflammatory processes [22,27,35,37,38,39]. In contrast, moderate-to-high thermal intensities, based on patient tolerance, were applied for chronic musculoskeletal conditions—such as osteoarthritis and tendinopathies—and vascular disorders to target fibrotic tissue and fluid accumulation [25,26,29,30,31,32,33,34].

Dosing and treatment duration varied considerably, ranging from a single session to evaluate immediate effects on pain and flexibility, to protocols of 12 to 18 sessions distributed over 3 to 8 weeks for chronic conditions. The average duration per session was 20 to 40 min. Regarding the application technique, manual dynamic application associated with mobilization or massage predominated. However, recent studies (e.g., Cau et al. [26], Uzun et al. [25]) introduced the use of automatic or static electrodes, particularly for the management of edema and limb volume.

#### 3.4.2. Comparators and Control Groups

To isolate the specific therapeutic effects of NIRF, the included trials employed diverse comparator strategies ranging from inert placebo interventions to active physical modalities.

A significant proportion of the studies implemented sham (placebo) interventions involving deactivated devices or “phantom” applications to blind participants and assessed the specific physiological effects of the radiofrequency current. This methodological design was utilized across various pathologies, including acute muscle injuries [22], knee osteoarthritis [33,34], post-surgical recovery [35], stroke-related spasticity [12,36], and diabetic neuropathy [38,39].

Regarding standard clinical practice, NIRF was frequently evaluated as an adjuvant to conventional care appropriate for each pathology. In musculoskeletal disorders, the control groups predominantly received therapeutic exercise protocols, orthopedic manual therapy, or patient education, as seen in the studies by Albornoz-Cabello et al., Davari et al., and Tezen et al. [27,29,30]. In the specific context of vascular pathologies, standard care consisted of compression therapy combined with structured walking pro-grams [25].

Finally, several trials assessed the efficacy of NIRF in direct comparison with other physical agents or established modalities. These active comparators included Transcuta-neous Electrical Nerve Stimulation (TENS) [28] and Ultrasound for musculoskeletal pain [23], Low-Level Laser Therapy (LLLT) for neuropathic conditions [37], and pressotherapy or Manual Lymphatic Drainage (MLD) for lymphedema management [26].

#### 3.4.3. Outcome Measures

To evaluate the efficacy of NIRF, the included studies utilized a heterogeneous array of instruments tailored to the specific pathology. Pain intensity was the most consistently reported primary outcome, assessed in almost all musculoskeletal and vascular trials. Specifically, the Visual Analogue Scale (VAS) was the predominant instrument [22,26,28,29,31,32], whereas Jang et al. [23] utilised the Numeric Rating Scale (NRS). Additionally, neuropathic pain characteristics were specifically evaluated using the DN4 (Douleur Neuropathique 4) questionnaire [29,31,32].

Regarding functional status and disability, the choice of instrument depended on the target condition. In knee osteoarthritis, the WOMAC (Western Ontario and McMaster Universities Osteoarthritis) Index was the gold standard for assessing pain, stiffness, and physical function [30,33,34]. For patellofemoral pain syndrome, pathology-specific scales such as the Kujala Score and the Lower Extremity Functional Scale (LEFS) were employed [29,31,32], while in sports-related groin pain, the HAGOS (Copenhagen Hip and Groin Outcome Score) and FASH questionnaires (Functional Assessment System for the Hip) were utilized to assess symptoms and participation in physical activities [22,28].

Lastly, complementing these patient-reported outcomes, several trials included quantitative physiological measurements. In neurological conditions, spasticity was graded using the Modified Ashworth Scale (MAS), and muscle tone was objectively quantified via myotonometry [12,24,36]. In parallel, for diabetic neuropathy, nerve function was assessed through Electroneuromyography (measuring Nerve Conduction Velocity) and sensory testing with Semmes-Weinstein monofilaments [37,38,39]. In the specific context of vascular disorders, therapeutic success was determined by changes in limb volume or circumference using tape measures or 3D laser scanners [25,26].

Regarding the timeframe of effects, the majority of the included studies focused on short-term outcomes, measured either immediately post-intervention or within a 4-to-8-week follow-up period. In fact, only one trial [31] provided mid-term data beyond 3 months. Consequently, the results of this meta-analysis primarily reflect the immediate and short-term therapeutic efficacy of NIRF, and its long-term sustainability remains to be established through future research.

#### 3.4.4. Synthesis of Effectiveness by Clinical Domain

The synthesis of effectiveness results is presented according to the clinical domains:

- Musculoskeletal Disorders: In the realm of acute sports injuries, NIRF demonstrated superior efficacy compared to conventional protocols. Specifically, Kelli et al. [28] reported faster recovery rates for hamstring injuries compared to TENS, while Nazari et al. [26] observed significant pain reduction in adductor-related groin pain compared to sham therapy. Conversely, in the case of acute ankle sprains, Davari et al. [27] found no significant difference between the intervention and standard care, suggesting that the efficacy of NIRF may be tissue-specific or dependent on the injury severity.

Shifting the focus to chronic knee pathologies, such as osteoarthritis and Patellofemoral Pain Syndrome (PFPS), the evidence was more consistent. The majority of studies [29,30,31,32,33,34] concluded that radiofrequency combined with therapeutic exercise was significantly superior to exercise alone or sham therapy, demonstrating consistent improvements in functional indices (e.g., WOMAC, LEFS) and pain scores (VAS). Notably, in post-surgical knee recovery, NIRF application was associated with reduced analgesic consumption and faster return to daily activities. However, it is worth noting that when compared to other deep heating modalities, the added value of NIRF was less distinct; for instance, Jang et al. [23] found no statistically significant difference between high-frequency NIRF (4.4 MHz) and ultrasound therapy in terms of pain reduction.

- Neurological Disorders: Interventions utilising capacitive and resistive transfer demonstrated significant potential for managing muscle tone and nerve function. In the context of central nervous system disorders, specifically in chronic stroke survivors, NIRF significantly reduced spasticity (hypertonia) and improved passive range of motion compared to sham interventions [12,24,36]. In parallel, for peripheral nervous system conditions such as diabetic neuropathy, NIRF, either alone or combined with Low-Level Laser Therapy, demonstrated superiority over control groups, significantly improving nerve conduction velocity and tactile sensation [37,38,39].
- Vascular and Lymphatic Disorders: Outcomes in the vascular domain focused on volume management and fluid dynamics. Both Uzun et al. [25] and Cau et al. [26], addressing lipedema and lymphedema, respectively, reported significant reductions in limb volume and circumference in favor of the radiofrequency groups compared to standard compression therapy or Manual Lymphatic Drainage (MLD) alone. Additionally, both studies observed significant improvements in quality of life scores and a reduction in the reported sensation of limb heaviness.

#### 3.4.5. Safety and Adverse Events

A crucial aspect of the synthesis was the evaluation of safety. Across the included studies, Non-Invasive Radiofrequency was reported as a safe intervention with a high safety profile. No serious adverse events (e.g., burns, skin damage, or exacerbation of symptoms) were recorded in the experimental groups. Mild and transient side effects, such as temporary erythema or excessive heat sensation during application, were rare and resolved immediately after adjusting the intensity or concluding the session. Consequently, the dropout rate related to the intervention was negligible across all clinical domains.

### 3.5. Quantative Synthesis (Meta-Analysis Results)

A meta-analysis was conducted to assess the effects of NIRFR on pain intensity and functional outcomes compared to control groups. A total of 13 studies were analyzed for pain intensity and 14 studies for functional outcomes. Following the methodology described in Section 2.6, data from the active control or standard care groups were prioritized for the analysis.

#### 3.5.1. Pain Intensity (VAS)

**Figure 4:**
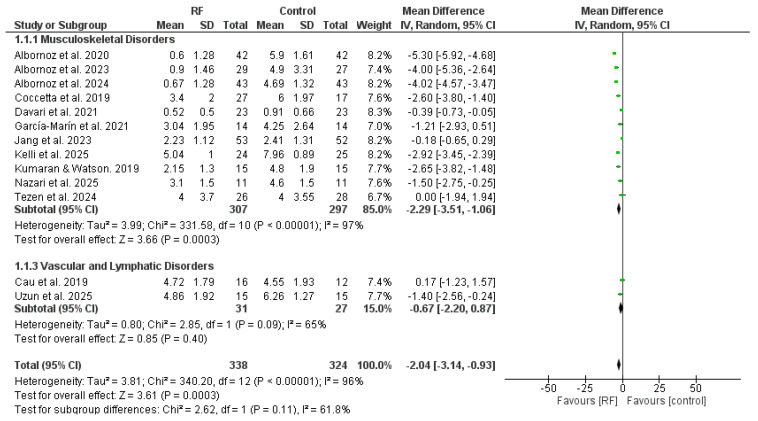
Forest plot comparing the effects of non-invasive radiofrequency (RF) versus control groups on pain intensity (VAS). All studies shown (Author, Year) are included in the study’s main reference list. Note: RF = Radiofrequency.

Data from 13 studies involving 662 participants (338 in the experimental group and 324 in the control group) were included. The results are presented according to the etiology of the disorder, divided into two subgroups: Musculoskeletal Disorders and Vascular and Lymphatic Disorders. The detailed forest plot is shown in Figure 4.

- Musculoskeletal Disorders: Eleven comparisons were analyzed in this subgroup. The analysis revealed a statistically significant difference in favor of the experimental group compared to the control group (MD = −2.29; 95% CI = −3.51 to −1.06; Z = 3.66, *p* = 0.0003).

This indicates that NIRF significantly reduces pain intensity in musculoskeletal conditions. However, considerable heterogeneity was observed (Chi^2^ = 331.58, I^2^ = 97%).

- Vascular and Lymphatic Disorders: Two studies (Cau et al. and Uzun et al. [25,26]) assessed the effects on vascular and lymphatic pathologies. In contrast to the musculoskeletal subgroup, no statistically significant difference was found between the experimental and control groups (MD = −0.67; 95% CI = −2.20 to 0.87; Z = 0.85, *p* = 0.40). The heterogeneity for this subgroup was substantial (I^2^ = 65%).
- Overall Analysis (Pain): When combining all subgroups, the global analysis favored the use of NIRF over the control group for pain reduction (MD = −2.04; 95% CI = −3.14 to −0.93; *p* = 0.0003). Nevertheless, these results should be interpreted with caution due to the high overall heterogeneity (I^2^ = 96%).

**Figure 5:**
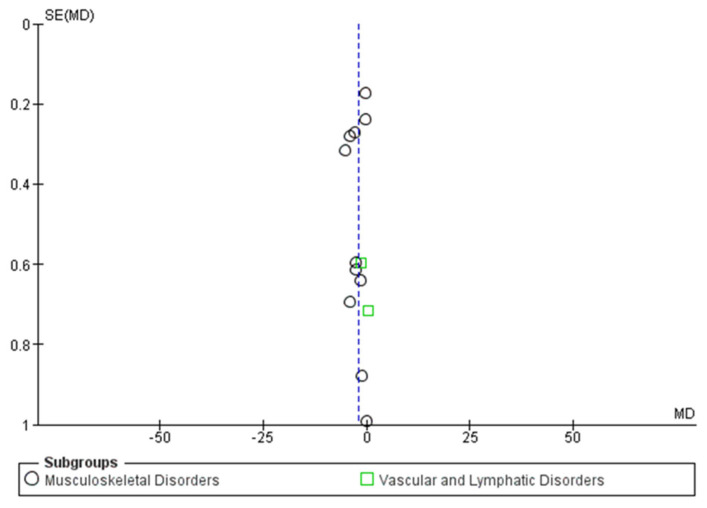
Funnel plot for pain. The blue dashed line represents the overall pooled effect size of the meta-analysis.

The risk of publication bias was assessed through a funnel plot (Figure 5) and statistically validated using Egger’s regression test. The visual distribution appeared relatively symmetrical, and the statistical analysis confirmed the absence of significant publication bias (*p* > 0.05). However, the overall analysis showed considerable statistical heterogeneity (I^2^ = 96%), which was further explored through sensitivity analysis (leave-one-out). The exclusion of any single study did not significantly alter the pooled effect size, nor did it substantially reduce heterogeneity, suggesting that the variance is likely due to the diverse clinical protocols and pathologies included.

#### 3.5.2. Functional Outcomes

**Figure 6:**
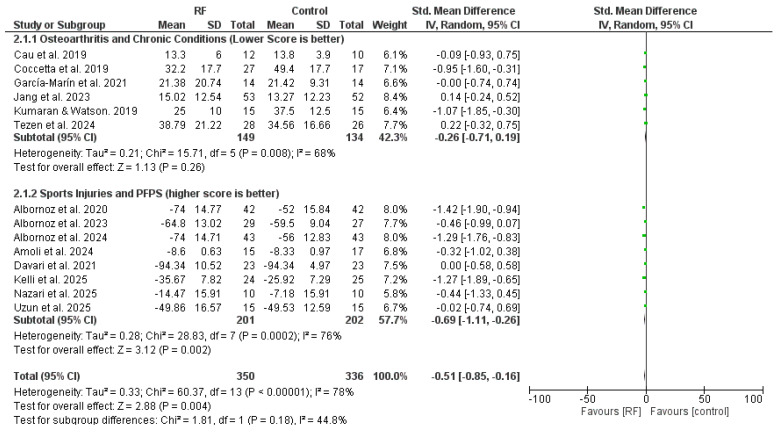
Forest plot comparing the effects of non-invasive radiofrequency (RF) versus control groups on functional outcomes. All studies shown (identified by Author and Year) are included in the study’s main reference list. Note: SMD = Standardized Mean Difference; RF = Radiofrequency.

Functional outcomes were assessed in 14 studies involving 686 participants (350 in the experimental group and 336 in the control group). The Standardised Mean Difference (SMD) was calculated due to the variety of instruments used, such as the Western Ontario and McMaster Universities Osteoarthritis Index (WOMAC), the Lower Extremity Functional Scale (LEFS), and the Timed Up and Go (TUG) test. The detailed forest plot is shown in Figure 6.

The overall analysis demonstrated a statistically significant improvement (SMD = −0.51; *p* = 0.004). Notably, the subgroup of ‘Sports Injuries and PFPS’ showed a more robust effect (SMD = −0.69) compared to chronic conditions. To account for the high heterogeneity (I^2^ = 78%), a random-effects model was maintained as the most conservative and appropriate approach. Statistical testing via Egger’s test showed no evidence of publication bias (*p* > 0.05).

Subgroup analysis revealed the following:

- Sports Injuries and PFPS: The intervention was statistically effective for this subgroup (SMD = −0.69; 95% CI: −1.11 to −0.26; *p* = 0.002), indicating a clear functional benefit in sports-related and patellofemoral conditions.
- Osteoarthritis and Chronic Conditions: The analysis for this subgroup did not reach statistical significance (SMD = −0.26; 95% CI: −0.71 to 0.19; *p* = 0.26). This suggests that functional improvements in chronic pathologies such as osteoarthritis may be more variable or less pronounced compared to acute sports injuries.

**Figure 7:**
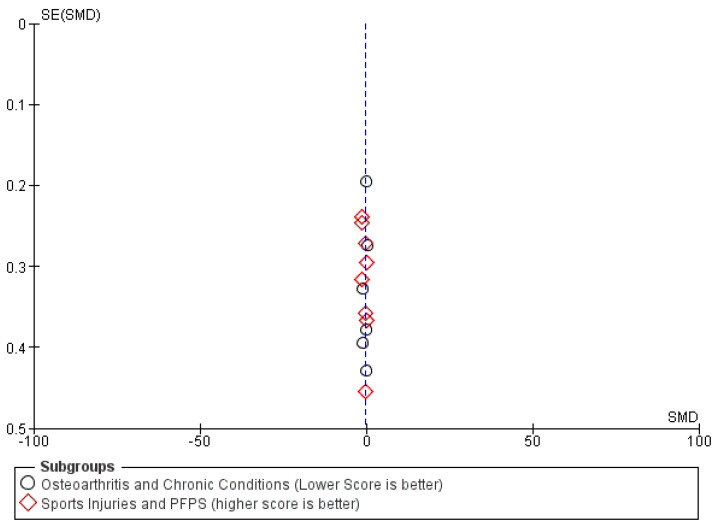
Funnel plot function. The blue dashed line represents the overall pooled effect size of the meta-analysis for functional outcomes.

Finally, visual inspection of the funnel plot (Figure 7) revealed a symmetrical distribution of studies, suggesting a low risk of publication bias.

#### 3.5.3. Analysis of Neurological Outcomes

A quantitative meta-analysis was attempted for the neurological subgroup based on the available studies. However, given the significant clinical heterogeneity (combining variables such as spasticity, nerve conduction velocity, and tactile sensation), methodological differences, and high statistical heterogeneity, pooling these data was deemed inappropriate. Consequently, the efficacy of NIRF in neurological conditions has been described qualitatively in the Narrative Synthesis (Section 3.4.4).

## 4. Discussion

This systematic review and meta-analysis provide a comprehensive synthesis of the efficacy and safety of Non-Invasive Radiofrequency across musculoskeletal, neurological, and vascular pathologies. To our knowledge, this is the first study to integrate evidence from these three distinct clinical domains, offering a holistic perspective on the therapy’s potential beyond a single specialty. The following discussion interprets these findings within the context of current literature, addressing the “therapeutic window,” biological mechanisms based on tissue type, and the comparative safety profile against invasive modalities.

### 4.1. Effectiveness and the "Therapeutic Window"

The primary finding of this review is that NIRF induces a statistically significant reduction in pain intensity and improvement in functional outcomes across a diverse range of musculoskeletal conditions. These results align with recent meta-analytic evidence, such as findings by Sadri, S et al., who reported significant standardized mean differences in pain reduction at 4 and 8 weeks [7], and Ribeiro et al., who highlighted its versatility across spinal and extremity pathologies [10]. Notably, the pooled mean difference observed for pain reduction (MD = −2.04) not only achieves statistical significance but also exceeds the established Minimal Clinically Important Difference (MCID) of 2 points. This indicates that the therapeutic effect of NIRF is perceivable and clinically meaningful for patients, transcending mere statistical probability.

However, beyond statistical significance, the clinical relevance of these findings appears to be closely linked to the intervention strategy. The analysis reveals an additive or synergistic effect: the majority of included studies compared NIRF combined with therapeutic exercise against exercise or standard care alone. The superior results in the experimental groups suggest that NIRF acts as a physiological facilitator, opening a “therapeutic window”. By reducing pain and modifying tissue viscoelasticity, as observed in the hamstring and adductor injury subgroups [22,28], NIRF likely enables patients to engage more effectively in rehabilitation programs.

This aligns with the concept that electrophysical agents should not be viewed as passive “magic bullets,” but rather as catalyzers that optimize the effects of mechanotherapy and active movement. This perspective reinforces the conclusion of Violan et al., who posited that NIRF is most beneficial when used as an adjunct to conventional physiotherapy protocols rather than a standalone passive treatment [11]. Furthermore, our subgroup analysis suggests that this “window” might be more effectively utilized in certain conditions. Specifically, the impact on functional status appears more robust in acute sports-related injuries (*p* = 0.002) than in chronic degenerative conditions like knee osteoarthritis, where functional scores did not reach statistical significance (*p* = 0.26). This suggests that NIRF might be optimized when targeting high-metabolism tissues or acute inflammatory phases. 

While the statistical significance was robust, particularly in acute injuries, the clinical relevance (magnitude of effect) appears to be dependent on the concurrent use of active therapy, reinforcing the need for multimodal approaches.

### 4.2. Hypothesized Mechanisms of Action

The synthesis of results suggests distinct therapeutic trends that may correlate with the target tissue’s biological state and metabolic activity. A statistically significant effect was observed in acute sports injuries (*p* = 0.002), which aligns with the meta-analysis by Kudich et al. (2025) [8]. While clinical commentaries [40] suggest that these interventions might enhance biomechanical parameters, direct evidence linking NIRF to improved running economy remains limited in the context of the trials reviewed.

Physiologically, the benefits observed in acute injuries have been theoretically attributed to strong thermal and non-thermal effects. De Sousa et al. [5] hypothesized that capacitive-resistive transfer increases oxyhemoglobin and enhances blood perfusion; however, since the included RCTs did not directly monitor microvascular changes, this remains a plausible but unconfirmed mechanism for the accelerated recovery observed. The discrepancy between the robust response in acute injuries and the less pronounced improvement in chronic osteoarthritis (*p* = 0.05) could suggest a “tissue responsiveness” factor, where well-vascularized muscle may respond more vigorously to electrical stimulation than hypovascular, degenerative cartilage.

In the neurological domain, improvements in nerve conduction velocity [37,38,39] support the hypothesis that NIRF might address ischemic components of neuropathy by influencing endoneural microcirculation. In the vascular and lymphatic domain, the results present a contrast between functional and analgesic outcomes. Although the meta-analysis found no significant difference in pain reduction (*p* = 0.40), the findings of Uzun et al. [25] suggest that analgesic benefits in lipedema may be transient. Similarly, the work of Cau et al. [26] indicates that while NIRF appears effective for volume reduction—potentially by modifying tissue viscoelasticity—its specific added value for pain relief is less distinct compared to multidisciplinary standard care. Therefore, it is hypothesized that the benefits observed in vascular and lymphatic conditions might be optimized through specific maintenance protocols; however, this remains a clinical conjecture that requires validation through long-term longitudinal studies.

### 4.3. Comparison with Invasive and Thermal Modalities

The findings of this review align with previous evidence supporting the use of non-invasive radiofrequency for musculoskeletal pain [7,8,10,41,42]. In the broader context of radiofrequency therapies, a recent meta-analysis has confirmed that invasive RF can yield medium to large effects in specific regions, such as the shoulder and sacroiliac joint, by aiming to interrupt or alter nociceptive pathways. However, results for other outcomes remain non-significant, and solid evidence regarding its broad effectiveness is still evolving [43]. Crucially, it is necessary to distinguish the non-invasive approach (NIRF) from these invasive interventions.

As reviewed by Bhatia et al., minimally invasive procedures like radiofrequency ablation (RFA) show high success rates in chronic hip pain by targeting sensory nerves, but they lack high-quality RCTs and are inherently destructive [44]. Similarly, invasive Pulsed Radiofrequency (PRF) is described as a non-ablative neuromodulatory technique; however, significant heterogeneity and a lack of standardized protocols continue to contribute to variable clinical outcomes [45]. Furthermore, limitations regarding high recurrence rates and insufficient long-term efficacy for conditions like radicular pain have also been observed [46].

In contrast to these invasive neural-targeting interventions, NIRF offers a regenerative and tissue-preserving alternative. While invasive RF primarily targets symptom suppression via neural inhibition or ablation [47], NIRF aims to improve tissue metabolism and circulation [34].

Furthermore, regarding safety, NIRF presents a superior profile compared to traditional thermal modalities. Unlike high-power shortwave or microwave diathermy, which carry risks of burns due to uncontrolled radiation and difficulty in focusing the energy precisely [48], NIRF utilizes a low-frequency current-controlled application (TECAR). This generates endogenous heat, allowing for precise thermal targeting of deep tissues without overheating the superficial layers [4]. This effect is explained by the biophysical principles of capacitive-resistive energy transfer, where the specific frequency allows for a reduction in skin impedance, favoring energy deposition in deeper, high-impedance tissues, such as tendons, ligaments, and joints, rather than the epidermal layer. This mechanism is supported by cadaveric evidence demonstrating that NIRF can significantly increase capsular and intra-articular temperatures through high-power protocols, while also maintaining current flow even in low-power, non-thermal applications [49]. 

This safety profile makes it particularly suitable for continuous rehabilitation and combination with active therapy. Indeed, evidence from randomized controlled trials suggests that adding radiofrequency to therapeutic exercise produces greater pain relief compared to exercise alone, highlighting its potential as a complementary intervention rather than a passive monotherapy [32]. While physical modalities like deep heat typically provide short-term analgesic effects, the integration of NIRF with active mechanotransduction offers a rationale for extending these benefits to functional recovery [6].

### 4.4. Interpretation of High Statistical Heterogeneity

A notable feature of this meta-analysis is the high statistical heterogeneity observed (I^2^ > 90%). Contrary to an interpretation of broad external validity, this degree of variability represents a significant methodological limitation that weakens the strength of the pooled effect estimates. While high heterogeneity is a frequent finding in rehabilitation literature, for instance, Kudich et al. (2025) [8] reported levels up to 98% and Sadri, S et al. observed (I^2^ > 86%) [7], its presence in this review indicates substantial inconsistency across the evidence base.

Our analysis pooled data from highly diverse clinical contexts, ranging from elite athletes with acute muscle tears to geriatric patients with chronic osteoarthritis. Furthermore, the included studies utilized a wide array of devices (e.g., Indiba, Winback, Capenergy), frequencies (448 kHz to 4.4 MHz), and application protocols. Despite this “clinical noise”, the risk of publication bias was statistically addressed. For the primary outcomes of pain and function, Egger’s regression test yielded non-significant results (*p* > 0.05), suggesting that the observed effects are not a result of small-study bias or selective publication [21].

Consequently, the results should be interpreted as a broad overview of the therapy’s potential rather than a precise clinical recommendation. Future guidelines must prioritize the establishment of standardized dosing to improve the reliability of clinical outcomes.

### 4.5. Limitations and Strengths

The interpretation of the present findings requires the consideration of several limitations inherent to the primary studies. First and foremost, the substantial statistical heterogeneity observed (I^2^ > 75%) persists even after conducting sensitivity analysis (leave-one-out), which indicates that no single study is responsible for the variance. Instead, this variability is likely attributable to the clinical diversity of the trials—utilizing different devices, emission modes, and dosimetries, which limits the strength of the clinical recommendations that can be derived.

Secondly, the inherent nature of thermal interventions poses a significant challenge for double-blinding. The sensory perception of heat makes it difficult to implement a true placebo (sham) control, inevitably introducing a high risk of performance bias across the majority of the included trials. Thirdly, although some studies incorporated follow-ups of up to 6 months [29], most of the evidence focuses on immediate or short-term outcomes (<3 months). Consequently, conclusions regarding the long-term sustainability of NIRF effects remain speculative and require further investigation.

Furthermore, a notable limitation is the inconsistent reporting of demographic data in some trials. As observed in the studies by Davari et al. [27] and Karas et al. [24], the absence of age and sex/gender descriptors hinders a full assessment of the findings’ generalizability. Given that age is a key factor influencing tissue healing and recovery rates, the lack of transparency in these variables restricts the ability to tailor clinical recommendations to specific age-related populations.

Regarding subgroup analysis, while results for lymphedema and neuropathy are promising, sample sizes were relatively small compared to the musculoskeletal group. Therefore, these specific findings should be interpreted as preliminary evidence rather than confirmed clinical efficacy [21].

Conversely, a major strength of this meta-analysis lies in the absence of significant publication bias, as statistically confirmed by Egger’s test (*p* > 0.05). The symmetrical distribution observed in the funnel plot suggests that studies with non-significant results were not systematically excluded. Finally, by strictly including only Randomized Controlled Trials, we have provided a synthesis of the highest level of evidence currently available in the field, despite the aforementioned clinical noise.

### 4.6. Clinical Implications

From a practical perspective, these findings support a paradigm shift regarding the application of electrophysical agents. The utilization of NIRF is advocated not as a standalone passive treatment, but rather as a preparatory or concurrent strategy to maximize tolerance and response to active rehabilitation. Consequently, the immediate analgesic and metabolic effects of the therapy may be leveraged to facilitate increased intensity or range of motion during therapeutic exercise, particularly in acute sports injuries.

Regarding vascular and lymphatic applications, given the potentially transient nature of the analgesic effects observed in conditions like lipedema, future research should explore whether specific maintenance protocols are necessary to sustain clinical improvements. At present, however, there is insufficient long-term evidence to support a formal recommendation for prolonged dosing, and this should be treated as a clinical hypothesis.

Furthermore, given the superior safety profile compared to invasive radiofrequency or uncontrolled thermal modalities, NIRF represents a valuable first-line conservative option before considering more aggressive interventions.

## 5. Conclusions

In conclusion, while Non-Invasive Radiofrequency appears to be a potential therapeutic option for managing pain and functional disability, the current evidence must be interpreted with significant caution due to the very high heterogeneity (I^2^ = 96%) and the lack of protocol standardization. The available data suggest a trend where NIRF may be most effective as a physiological facilitator when combined with therapeutic exercise, particularly in musculoskeletal conditions such as acute sports injuries and knee pathology. However, its efficacy in neurological and vascular domains, although promising regarding spasticity and limb volume, remains limited by small sample sizes and clinical variability. Given that most studies focus on short-term outcomes, there is an urgent need for high-quality, large-scale RCTs to standardize dosimetry and evaluate the long-term sustainability of these therapeutic effects beyond three months.

## Figures and Tables

**Table 1:** Characteristics and results of included studies.

Author Year Country Pathology	N Gender Age	Intervention Sessions Duration	Outcomes (Measuring Instruments)	Results
Musculoskeletal Disorders				
Acute Sports Injuries				
Kelli et al., 2025 [28] Greece Acute hamstring injury in athletes	N = 75 EG1: 24 EG2: 26 CG: 25 38 F/37 M 24.37 ± 7.70	EG1: Capacitive TECAR therapy Device: Winback^®^ (France) Frequency: 500 kHz Intensity: 40–60% (heat tolerance–based) Active electrode: circular, 6 cm, over injured area Passive electrode: 20 × 15 cm on anterior thigh Position: prone Technique: slow circular movements Dose: 1 session, 20 min EG2: TENS (80 Hz, 15–45 mA) 1 session, 20 min CG: Rest + RICE protocol 1 session, 20 min	Pain (VAS) Function (FASH) Flexibility (SLR, STR)	Pain: Greater VAS reduction with TECAR compared to TENS and rest at all post-intervention time points (*p* < 0.05) Percentage reduction: – TECAR: 38.75–63.33% – TENS: 16.67–25.00% – Rest: 2.81–9.81% Effect size: small–moderate (η^2^ ≈ 0.008) Function: Greater FASH improvement with TECAR vs. rest at 24 and 48 h (*p* < 0.05) TECAR showed larger percentage improvements than TENS and rest Flexibility: Greater improvements in SLR and STR with TECAR vs. TENS and rest Effect sizes small (η^2^ ≈ 0.001–0.002) No adverse events reported
Nazari et al., 2025 [22] Iran Adductor-related groin pain in athletes	N = 22 EG: 11/CG: 11 0 F/22 M	EG: TECAR therapy (WINBACK 3SE, France) Mode: capacitive + resistive Intensity: 30–40% Capacitive: 7.5 min Resistive: 7.5 min Total TECAR time: 15 min/session Frequency: 10 sessions Co-intervention: treadmill warm-up + adductor stretching CG: Sham TECAR (intensity 0) Same protocol, duration, and stretching exercises	Pain intensity (VAS) Hip range of motion: abduction/adduction (goniometer) Hip abductor/adductor strength (handheld dynamometry) Function and symptoms (HAGOS questionnaire, 6 subscales)	Pain (VAS): Significant reduction after 5 sessions (*p* = 0.01; d = −1.09) Greater reduction after 10 sessions (*p* = 0.001; d = −2.15) Effect maintained at 1-month follow-up (*p* = 0.001; d = −1.96) indicating large effect sizes ROM: Significant improvement in hip adduction ROM at 1-month follow-up (*p* = 0.03; d = 0.91) Strength and HAGOS: No significant between-group differences (small to moderate effect sizes) Adverse events: none reported
Davari et al., 2021 [27] Iran Acute Lateral Ankle Ligament Sprain (Athletes)	N = 46 EG: 23 CG: 23 Gender: NR Age. NR	EG: Standard PT (Ultrasound, TENS, Infrared/Cryotherapy) + TECAR: Intensity 10–30% (Low intensity, consistent with acute phase treatment) Frequency (Hz), Mode (Cap/Res), and Duration (min): NR CG: Standard PT 12 sessions	Pain (VAS) Swelling (Tape measure) ROM (Goniometer) Function (FAAM and ADL & Sport subscales)	Intra-group (Time): Both groups showed significant improvement in Pain, Swelling, ROM, and Function after 6 and 12 sessions (*p* < 0.001). Inter-group (Comparison): No significant difference was found between the TECAR group and Control group for any variable at any time point (*p* > 0.05). Both interventions were effective, but TECAR did not demonstrate superior efficacy over standard care in this study.
Knee Pathology				
Albornoz-Cabello et al., 2024 [29] Spain PFPS	N total = 86 GE (RF + exercise): 43 (17M/26F), 42 ± 4.3 yrs GC (exercise): 43 (17M/26F), 42 ± 4.3 yrs	GE: Supervised therapeutic exercises + neuromodulation via monopolar dielectric radiofrequency (ABD Modular^®^, Biotronic Advance, Spain), pulsed 30 V, 640–830 kHz, dynamic rotation/translation of anterior knee, 5 mL almond oil coupling medium, 12 min/session. GC: Same supervised therapeutic exercises as GE, without RF. GE: 10 sessions RF + 15 exercise sessions RF: Week 1: 5 sessions (Mon–Fri), Week 2: 3 sessions (Mon, Wed, Fri), Week 3: 2 sessions (Mon, Thu) Exercise: Daily Mon–Fri for 3 weeks	Pain (VAS) Lower extremity function (%LEFS) Patellofemoral function: Kujala (% Kujala score) Knee ROM: flexion & extension (goniometer)	GE: ↓VAS significantly vs. GC, η^2^ = 0.56, maintained at 6 months LEFS: improved ≥9 points, η^2^ = 0.17, maintained Kujala: improved ≥9.5 points, η^2^ = 0.14, maintained Flexion ROM improved, η^2^ = 0.37; Extension: no significant difference No adverse events; analgesic use unchanged
Tezen et al., 2024 [30] Turkey Knee Osteoarthritis	N = 54 GE = 28 CG = 26 (60.5 ± 8.2 yrs) 30 F/24 M 61.5 ± 10.0	GE: CPT + TECAR CPT (TENS: 80 Hz, 20 min; Hot Pack: Superficial heat, 20 min; Exercise: Knee isometric and TheraBand exercises) Frequency: 5 sessions/week for 2 weeks (10 sessions of CPT). GC: CPT	Pain: VAS (during Rest, Motion, Night). Function/Disability: WOMAC Index. Isometric Strength Quadriceps (Diers Myoline System) ROM (flex/ext) (Goniometer) Measured at Pre, Post, 1 month, and 3 months.	Within-Group: Significant improvements in VAS, WOMAC, and Quadriceps Strength in both groups at all follow-ups (*p* < 0.05). Between-Group: No significant difference found between groups for most variables. Exception: Group 2 (TECAR) had significantly lower Night Pain at 1 month vs. Group 1 (*p* = 0.034). TECAR did not provide superior benefit over conventional therapy for function or strength, only short-term night pain relief.
Albornoz et al., 2023 [31] Spain PFPS	N = 56 EG: 27 11 F/16 M; 42.3 ± 15.5 CG: 29 18 F/11 M; 51.0 ± 10.9 yrs	EG: Diathermy (radiofrequency) + therapeutic exercise CG: Therapeutic exercise alone (concentric & eccentric quadriceps strengthening, hamstring bridges, gluteus medius clam, gastrocnemius–soleus stretching) 10 diathermy sessions Exercise: daily (Mon–Fri) 3 weeks Follow-up: 6 months	Pain (VAS, Neuropathic Pain) Function (LEFS, Kujala) ROM: Flex/Ext (goniometry)	VAS: 4.0 reduction in experimental vs. 0.9 in control (d = 1.45, *p* < 0.001) DN4: 4.1 → 0.2 in experimental vs. 3.8 → 1.9 in control (d = 1.86, *p* < 0.05) Kujala: 54.4 → 73.7 vs. 49.5 → 69.2 (not significant) LEFS: 42.3 → 64.8 vs. 45.3 → 59.5 (not significant) Flexion ROM: 118.3° → 133.9° vs. 117° → 125.9° (d = 0.70, *p* < 0.05) Extension ROM: 1.2° → 0.2° vs. 0.5° → 0.9° (not significant)
Jang et al., 2023 [23] South Korea Knee Osteoarthritis	N total = 105 RF: 53 (1M/52F), 61.5 ± 5.1 yrs US: 52 (48F/4M), 60.9 ± 5.1 yrs	RF group: Transcutaneous 4.4 MHz RF diathermy (HIPER-500^®^, 45 W/cm^2^ output, CET + RET, moving probe, gel coupling, 15 min/session) US group: Therapeutic ultrasound 3 MHz, max 1.5 W/m^2^, moving probe, 15 min/session 10 sessions over 4 weeks, 3 sessions/week (both groups)	Pain: NRS (0–10) Function: WOMAC total and subscales, Lequesne Index Gait: stride length, stride velocity, cadence QoL: SF-36 Adverse effects	Both groups: significant ↓pain (NRS), improved WOMAC & Lequesne vs. baseline RF: significant ↑cadence; US: not significant Both groups: gait improvements at 4-week follow-up SF-36: improvements in bodily pain (RF) and general health/social functioning (US) No significant differences between groups at any time point No adverse effects reported
Albornoz-Cabello et al., 2020 [32] Spain PFPS	N = 84 EG: 42/CG: 42 52F/32M Mean age: 50 ± 13.4 years	EG: Monopolar Dielectric Radiofrequency (MDR) Device: ABD Modular^®^ (Biotronic Advance Develops^®^, Spain) Frequency: 840 kHz Voltage: 30 V Pulsed emission Dynamic application (rotational and translational movements) over the anterior knee surface Dielectric medium: almond oil (5 mL) Duration: 12 min/session Frequency: 10 sessions over 3 weeks (5 + 3 + 2) Co-intervention: daily home-based exercise program (10 min/session; stretching, cycling, and load-management advice) CG: Daily home-based exercise program only (10 min/session)	Pain: VAS, DN4 Function: LEFS, Kujala score Knee ROM: flexion and extension (goniometer) Analgesic drug intake	Pain: Significant VAS reduction (*p* < 0.001; η^2^ = 0.67 → large effect) Significant DN4 reduction (*p* < 0.001; η^2^ = 0.66 → large effect) Function: LEFS improvement (*p* < 0.001; η^2^ = 0.45 → large effect) Kujala score improvement (*p* < 0.001; η^2^ = 0.39 → large effect) ROM: Increased knee flexion (*p* < 0.001; η^2^ = 0.64 → large effect) Increased knee extension (*p* = 0.009; η^2^ = 0.08 → moderate effect) No significant changes in analgesic intake No adverse events reported
Coccetta et al., 2019 [33] Italy Knee osteoarthritis	N = 53 EG: 31 (6 M/25 F) GC: 22 (0 M/22 F) Age: 44–84 years	EG: CRET (Capacitive and Resistive Electric Transfer, Tecar therapy) Device: Tecar Unibell HCR 902^®^ Quadriceps and peripatellar region Capacitive: 40–60 VA/5 min Resistive: 30–50 W/10 min Capacitive: 40–60 VA/5 min GC: Sham CRET (0 W/VA), same positioning and duration, no exercise 6 sessions (3/week, 2 weeks) Duration per session: 20 min	Pain, stiffness, function: WOMAC (primary) Pain: VAS (secondary) Quadriceps strength: MRC (secondary) Evaluation: T0, T1, T2 (1 month), T3 (3 months)	EG: ↓ WOMAC total (pain, stiffness, function) at T1, T2, T3 vs. T0 ↓ VAS at all follow-ups ↑ Quadriceps strength (MRC) at T2 and T3 Clinically meaningful changes (MCID >15 WOMAC) GC: No significant changes in WOMAC or VAS ↓ Quadriceps strength at follow-ups Adverse events: none Effect persistence: up to 3 months
Kumaran & Watson. 2019 [34] United Kingdom Knee osteoarthritis	N total = 45 GE (CRMRF): 15 (6M/9F), 63 ± 10 yrs CE (Sham CRMRF): 15 (6M/9F), 63 ± 10 yrs GC: 15 (6M/9F), 60 ± 6.2 yrs	GE (Active CRMRF): Capacitive resistive monopolar radiofrequency (448 kHz)—Indiba Activ 902, 5 min CAP + 10 min RES, whole knee, emphasis on symptomatic areas, moderate heating perception CE (Sham CRMRF): Same procedure but no energy delivery GC (Standard care): Home exercises and standard physiotherapy	Pain: VAS (24 h) Function: WOMAC (pain, stiffness, function) Walking ability: Timed Up and Go (TUG) Knee ROM: goniometry Evaluation: baseline, post-treatment (4 wk), 1-month follow-up (8 wk), 3-month follow-up (16 wk)	GE: ↓ VAS, improved WOMAC post-treatment & 1-month follow-up (clinically significant), no meaningful changes in TUG or ROM CE: no clinically relevant changes GC: minimal improvements, not significant Adverse events: none reported Effect persistence: up to 1 month post-treatment Effect size: VAS g = 1.3–1.5, WOMAC g = 0.94
Post-Surgical Interventions				
García-Marín et al., 2021 [35] Spain Total Knee Replacement	N = 42 EG:14 Placebo Group (Sham):14 CG: 14 27 F/15 M 69.4 ± 7.2	EG: Exercise + TECAR (ABD Modular). 840 kHz (mod 140 kHz). 70% Pulsed Emission (Athermal/Low thermal), 30 V. 12 min. Anterior/Posterior knee. 5/week, 2 weeks, 10 sessions Placebo Group (Sham): Exercise + Sham Device. 12 min. 10 sessions CG: Exercise 30 min protocol: Active Knee Flex/Ext (10 min) + Walking (10 min) + Knee Ext with 5 kg weight (10 min). 10 sessions	Pain (VAS) Function (WOMAC, TUG, FSST) Quality of life (SF-12) Measured at Baseline and Post-treatment.	Pain: Significant interaction (*p* = 0.009). EG showed superior pain reduction vs. Placebo and CG (Large effect, Cohen’s d = 2.15). WOMAC: Significant interaction (*p* = 0.021). EG showed greater functional improvement. No significant difference: TUG, FSST, or SF-12 (*p* > 0.05) between groups, though EG improved significantly in TUG intra-group.
**Neurological Disorders**				
Chronic Stroke (Spasticity)				
García-Rueda et al., 2025 [12] Spain Chronic stroke with lower limb hypertonia	N = 36 EG: 18 (10 M/8 F) CG: 18 (10 M/8 F) Mean age: 58.6 ± 11.3 years	EG: TECAR therapy + functional massage Device: T-Plus Wintecare (Chiasso, Switzerland) • Resistive mode: 80–140 W Capacitive mode: 180–200 VA Application: gastrocnemius and quadriceps Duration: 1 session, 30 min CG: Sham TECAR (0 W/VA) + functional massage Same duration and steps	Hypertonia: MAS (hip, knee, ankle) Gait: 4-MWT Knee function: Fugl-Meyer (item IV) Weight-bearing ankle dorsiflexion: ALT Lower limb functional strength: 5TSTS	↓ Hypertonia (MAS) in knee and ankle only in EG ↑ Weight-bearing ankle dorsiflexion (ALT) in EG; effect sizes η^2^ = 0.02–0.17 (small–moderate) No significant changes in gait (4-MWT) or functional strength (5TSTS) No adverse events
Karas et al., 2024 [24] Poland Chronic stroke with lower limb hypertonia	N, gender, age = NR	GE: Single session of TECAR + Manual Therapy. GC: Control group (details not specified).	Spasticity (MAS) Neuromuscular properties (Myotonometry, Gastrocnemius tension) Muscle Strength (Dynamometer. Passive) PROM): Inclinometer.	Significant ↓ in Gastrocnemius tension (GE). Significant ↓ in MAS (Ankle dorsiflexion). Significant ↑ in Ankle ROM and Gastrocnemius PROM. Pain: TECAR was more effective for musculoskeletal pain vs. Control.
García-Rueda et al., 2023 [36] Spain Chronic stroke with lower limb hypertonia	N = 36 GE: n = 18 (11M/7F) GC: n = 18 (11M/7F) 58.5 ± 11.5	GE: Single 30 min session combining Functional Ma- ssage (FM) + TECAR (T-Plus, Wintecare). 1. Lumbar: Res (80–100 W), 7 min. 2. Hamstrings: Res (100–120 W), 5 min. 3. Gastrocnemius: Res (110–120 W), 5 min + Cap (180–200 VA), 4 min. 4. Quadriceps: Res (110–140 W), 5 min + Cap (180–200 VA), 4 min. GC: Identical FM protocol with Sham TECAR (device off, electrode pre-heated for blinding).	Myotonometry: No significant changes in objective neuromuscular properties (stiffness/tone) in either group. Muscle Tone: Modified Ashworth Scale (MAS) score. MAS Degrees: Goniometry. Passive ROM (PROM): Inclinometer. Neuromuscular properties: MyotonPro (Tone, Stiffness, Relaxation). Measured at T0 (baseline), T1 (immediate), T2 (30 min post).	MAS Score: Significant ↓ in Ankle Dorsiflexion for GE at T1 (*p* = 0.046) and T2 (*p* = 0.019). No change in Knee/Hip scores. MAS Degrees: GE improved significantly in Hip, Knee, and Ankle (*p* < 0.05). PROM: GE showed significant ↑ in Knee flexion (*p* = 0.012) and Ankle dorsiflexion (*p* = 0.034) vs. Control. Myotonometry: No significant changes in objective neuromuscular properties (stiffness/tone) in either group. Effect sizes (η^2^) > 0.14 (large) for clinical variables.
Diabetic Peripheral Neuropathy				
Amoli et al., 2024 [37] Iran Diabetic Peripheral Neuropathy	N = 45 GE1: 15 GE2: 15 GC: 15 65.7 ± 7.6 30 F/15 M	GE1: TECAR+ exercise TECAR + Sham LLLT + Exercise. Details: Same TECAR protocol (Spine + Knee). Laser turned off. Freq: 10 sessions. GE2: Combined (TECAR + Laser) + exercise TECAR + LLLT + Exercise. TECAR: 250/500 kHz. Int: 40–50% (Athermal). Site: Lumbosacral (25 min) + Popliteal (25 min). LLLT: 890 nm/630 nm (12 min). Freq: 10 sessions. GC: Laser Only (Active Control)+ exercise LLLT + Sham TECAR + Exercise. Details: Active Laser. TECAR intensity 0%. Freq: 10 sessions. Exercise: 15 min of therapeutic exercises (Strengthening, Stretching, and Weight-bearing) 3/week, 10 sessions	Tibial Nerve Conduction Velocity (Electroneuromyography) Sensory Nerve (Sural Nerve Amplitude) Sensation (Semmes-Weinstein Monofilament) Vascular (Ankle-Brachial Index) Measured at Pre, Post (10 sessions), and 3-month Follow-up.	Post-Treatment (10 sessions): All groups improved. GE2 (Combined) showed significant superiority in Tibial MNCV vs. GC. 3-Month Follow-up: GE2 (Combined): Superior to GC in all variables (*p* < 0.05). GE1 (TECAR): Superior to GC (Laser) in Sensory outcomes (Sensation & SNA). TECAR (alone or combined) maintains sensory benefits better than Laser alone.
Niajalili et al., 2023 [38] Iran Diabetic Peripheral Neuropathy	N = 24 EG: 12 CG: 12 60.4 ± 8.9	EG: IR + Active TECAR CG: IR + Sham TECAR IR on foot surfaces (870 nm, 30 min) TECAR on tibial nerve (20 min) 10 sessions (3/week)	Neuropathy Symptoms (MNSI) Tibial Nerve Conduction Velocity (Electroneuromyography) Measured at Baseline, Post-treatment (10 sessions), and 6-week Follow-up.	Neuropathy Symptom: Post-treatment: Significant improvement in EG vs. CG (*p* < 0.001). 6-week Follow-up: Improvement maintained only in EG (*p* < 0.05). Tibial Nerve Conduction Velocity: Post-treatment: Significant increase in both groups (*p* < 0.001). 6-week Follow-up: Physiological improvement maintained only in Experimental group (*p* < 0.05). Overall: Significant Time × Group interaction (MNSI: F = 30.59; TNCV: F = 79.39; *p* < 0.001). Effect sizes not explicitly reported, but high F-values indicate strong statistical significance.
Niajalili et al., 2020 [39] Iran Diabetic Peripheral Neuropathy	N = 24 EG: 12 CG: 12 60.04 ± 8.88	EC: (IR + Active TECAR) CG: IR + Sham TECAR IR: 870 nm wavelength, density 1.3 j/cm^2^/, 30 min duration TECAR: Capacitive, Tibial nerve pathway, <50% intensity (non-thermal/low-thermal), 20 min	Pain: VAS (10 cm). Tactile Sensation: Semmes-Weinstein monofilament (5.07/10 g) Measured pre- and post-treatment (after 10 sessions)	Pain: Significant reduction in both groups, but significantly greater reduction in Exp vs. Control (Post-test VAS: 0.79 vs. 2.17; *p* = 0.002) Tactile Sensation: Significant improvement in both groups, but significantly greater improvement in Exp vs. Control (*p* < 0.001) TECAR added significant value over IR alone
**Vascular and Lymphatic Disorders**				
Uzun et al., 2025 [25] Turkey Lipedema	N = 30 EG: 15 CG: 15 49.3 ± 13.2	EC: TECAR + Standard Care) CG: Standard Care only TECAR Therapy (BTL-6000 TR-Therapy PRO). Frequency ~500 kHz. Intensity adjusted to pleasant warmth. Capacitive (5 min): Superficial tissues (skin, subcutaneous fat). Resistive (10 min): Deeper fibrotic/connective tissues. Total: 15 min/session. Frequency: 2 sessions/week for 3 weeks (Total 6 sessions). Control Modality: Standard Care only. Compression: Class II leggings (23–32 mmHg), worn ≥ 8 h/day. Exercise: Structured walking program, moderate intensity (Borg 11–13), 20 min, 3 times/week. Frequency: Continuous management over the study period	Limb Circumference: (Mid-thigh, pretibial, supramalleolar) Pain (VAS). Function: (LEFS) Quality of Life (LYMQOL-Leg.) Measured at Baseline, 1 month, and 3 months.	Circumference: TECAR showed greater reduction in all regions at 1 month (*p* < 0.05). At 3 months, only supramalleolar reduction was maintained (*p* < 0.05). Pain (VAS): Significant ↓ in TECAR at 1 month (*p* = 0.003), but effect was not maintained at 3 months. Change was significantly better vs. Control at both time points.
Cau et al., 2019 [26] Italy Lower limb lymphedema	N = 48 EG: 12 CG: 12 48 F 63.0 ± 14.2	All 4 groups multidisciplinary rehabilitation program (diet, adapted physical activity, physiotherapy, psychological support). TECAR Group Rehab + TECAR Therapy (CIM 200). 0.8–1.2 MHz, capacitive, medium-high power. 45 min/leg (15’ groin, 15’ popliteal, 15’ foot) 6 sessions Pressure Group Rehab + Pressure Therapy. (Distal-proximal gradient, 40 mmHg, 30 s cycles. 45 min/session) 6 sessions MLD Group Rehab + Manual Lymphatic Drainage (Vodder technique: slow, rhythmic, mild-intensity pressure. 60 min/session) Control Group Multidisciplinary rehabilitation program only 6 days/week for 4 weeks.	Limb Volume (3D Laser Scanner LS3D) Functional mobility (Timed Up and Go-TUG) Pain/Heaviness (VAS)	Volume: TECAR showed significant reduction in whole limb (9.7→9.4 dm^3^) and thigh (3.5→3.3 dm^3^) after 6 sessions (*p* < 0.05). Function/Pain: TUG and VAS improved significantly in all groups. Comparisons: Pressure and MLD showed less marked/localized volume reductions. Control showed no volume change.

Abbreviations: EG: experimental group; CG: control group; VAS: visual analogue scale; FASH: Functional Assessment Scale for Acute Hamstring Injuries; SLR: straight leg raise; STR: Sit-and-Reach Test; PFPS: patellofemoral pain syndrome; Mon: Monday; Fri: Friday; Wed: Wednesday; Thu: Thursday; CE: comparator group; Flex: flexion; Ext: extension; F: female; M: male; IR: infrared radiation; NR: not reported; MAS: Modified Ashworth Scale; PROM: passive range of motion; PT: physiotherapy; FAAM: Foot and Ankle Ability Measure; MNSI: Michigan Neuropathy Screening Instrument; CPT: conventional physical therapy; MNCV: nerve conduction velocity; LLLT: low-level laser therapy; FSST: five times sit-to-stand test.

**Table 2:** Methodological quality assessment (PEDro Scale).

Author, (Year)	Item 1	Item 2	Item 3	Item 4	Item 5	Item 6	Item 7	Item 8	Item 9	Item 10	Item 11	Total
Kelli et al., 2025 [28]	1	1	1	1	0	0	1	1	0	1	1	8/10
Nazari et al., 2025 [22]	1	1	1	1	1	0	1	1	1	1	1	9/10
Davari et al., 2021 [27]	1	1	0	1	0	0	1	1	0	1	1	6/10
Albornoz-Cabello et al., 2024 [29]	1	1	1	1	0	0	1	1	1	1	1	8/10
Tezen et al., 2024 [30]	1	1	1	1	0	0	1	1	1	1	1	8/10
Albornoz et al., 2023 [31]	1	1	1	1	0	0	1	1	0	1	1	7/10
Jang et al., 2023 [23]	1	1	1	1	1	0	1	1	0	1	1	8/10
Albornoz-Cabello et al., 2020 [32]	1	1	1	1	0	0	1	1	1	1	1	8/10
Coccetta et al., 2019 [33]	1	1	1	1	1	0	1	0	1	1	1	8/10
Kumaran & Watson. 2019 [34]	1	1	1	1	1	0	0	1	1	1	1	7/10
García-Marín et al., 2021 [35]	1	1	1	0	1	0	1	1	1	1	1	7/10
García-Rueda et al., 2025 [12]	1	1	1	1	0	1	1	1	1	1	1	9/10
Karas et al., 2024 [24]	1	1	1	1	0	1	1	1	1	1	1	9/10
García-Rueda et al., 2023 [36]	1	1	1	1	0	1	1	1	1	1	1	9/10
Amoli et al., 2024 [37]	1	1	1	1	0	1	1	1	1	1	1	9/10
Niajalili et al., 2023 [38]	1	1	0	1	1	0	0	1	1	1	1	7/10
Niajalili et al., 2020 [39]	1	1	0	1	1	0	0	1	1	1	1	7/10
Uzun et al., 2025 [25]	1	1	1	1	0	0	0	1	1	1	1	7/10
Cau et al., 2019 [26]	1	1	1	1	0	0	0	1	1	1	1	7/10

“0” indicates those items that do not score; “1” indicates those items that score. Total score calculated out of 10; Item 1 is excluded from the total.

**Table 3:** Synthetic comparative summary of NIRF protocols.

Author (Year)	Clinical Condition	Frequency (kHz/MHz)	Mode (Cap/Res)	Duration (min/Session)	Total Sessions	Intensity/Dose
Kelli et al., 2025 [28]	Acute hamstring injury	500 kHz	Capacitive	20 min	1 session	Thermal (40–60%)
Nazari et al., 2025 [22]	Adductor groin pain	NR	Cap + Res	15 min	10 sessions	30–40% intensity
Davari et al., 2021 [27]	Acute ankle sprain	NR	NR	NR	12 sessions	10–30% (Athermal)
Albornoz-Cabello et al., 2024 [29]	PFPS	640–830 kHz	Monopolar	12 min	10 sessions	30 V Pulsed (Athermal)
Tezen et al., 2024 [30]	Knee Osteoarthritis	NR	NR	NR	10 sessions	NR
Albornoz et al., 2023 [31]	PFPS	NR	NR	NR	10 sessions	NR
Jang et al., 2023 [23]	Knee Osteoarthritis	4.4 MHz	Cap + Res	15 min	10 sessions	Thermal (45 W/cm^2^)
Albornoz-Cabello et al., 2020 [32]	PFPS	840 kHz	Monopolar	12 min	10 sessions	30 V Pulsed (Athermal)
Coccetta et al., 2019 [33]	Knee Osteoarthritis	NR	Cap + Res	20 min	6 sessions	Thermal (40–60 VA)
Kumaran & Watson., 2019 [34]	Knee Osteoarthritis	448 kHz	Cap + Res	15 min	12 sessions	Moderate heating
García-Marín et al., 2021 [35]	Knee Replacement	840 kHz	NR	12 min	10 sessions	30 V Pulsed (Athermal)
García-Rueda et al., 2025 [12]	Stroke (Spasticity)	NR	Cap + Res	30 min	1 session	High (180–200 VA)
Karas et al., 2024 [24]	Stroke (Spasticity)	NR	NR	NR	1 session	NR
García-Rueda et al., 2023 [36]	Stroke (Spasticity)	NR	Cap + Res	30 min	1 session	High (80–200 VA/W)
Amoli et al., 2024 [37]	Diabetic Neuropathy	250/500 kHz	NR	50 min	10 sessions	40–50% (Athermal)
Niajalili et al., 2023 [38]	Diabetic Neuropathy	NR	NR	20 min	10 sessions	NR
Niajalili et al., 2020 [39]	Diabetic Neuropathy	NR	Capacitive	20 min	10 sessions	<50% (Athermal)
Uzun et al., 2025 [25]	Lipedema	500 kHz	Cap + Res	15 min	6 sessions	Pleasant warmth
Cau et al., 2019 [26]	Lymphedema	0.8–1.2 MHz	Capacitive	45 min	6 sessions	Medium-High power

NR: Not Reported; Cap: Capacitive; Res: Resistive.

## Data Availability

The data supporting the findings of this study are available from the corresponding author upon reasonable request.

## Supplementary Materials

The following supporting information can be downloaded at https://www.mdpi.com/article/10.3390/jcm15062428/s1: Supplementary S1: PRISMA 2020 Checklist. Supplementary S2: Search strategy.

## References

[1] Ferrari A.J., Santomauro D.F., Herrera A.M.M., Shadid J., Ashbaugh C., Welsh S.D., Abate Y.H., Abbafati C., Abbas J., GBD 2021 Diseases and Injuries Collaborators (2024). Global incidence, prevalence, years lived with disability (YLDs), disability-adjusted life-years (DALYs), and healthy life expectancy (HALE) for 371 diseases and injuries in 204 countries and territories, 1990–2021: A systematic analysis for the Global Burden of Disease Study 2021. Lancet.

[2] Cohen S.P., Vase L., Hooten W.M. (2021). Chronic pain: An update on burden, best practices, and new advances. Lancet.

[3] Cieza A., Causey K., Kamenov K., Hanson S.W., Chatterji S., Vos T., Al Haboubi M., Badii M., Banigo A., Bark-er-Collo S. (2020). Global estimates of the need for rehabilitation based on the Global Burden of Disease study 2019: A systematic analysis for the Global Burden of Disease Study 2019. Lancet.

[4] Kumaran B., Watson T. (2015). Thermal build-up, decay and retention responses to local therapeutic application of 448 kHz capacitive resistive monopolar radiofrequency: A prospective randomised crossover study in healthy adults. Int. J. Hyperth..

[5] De Sousa-De Sousa L., Valera-Garrido F., Hernández-Guillen D., Sánchez-Atta M.A., Teeroovengadum V., Mina-ya-Muñoz F., Garcia-Soto A., Garcia-Moreno J.M., Morales-Asencio J.M., Luque-Suarez A. (2021). Application of Capacitive-Resistive Electric Transfer in Physiotherapeutic Clinical Practice and Sports. Int. J. Environ. Res. Public Health.

[6] Clijsen R., Leoni D., Kerkhofs A., Schneebeli A., Cescon C., Soldini E., Li L., Barbero M. (2020). Does the Application of Tecar Therapy Affect Temperature and Perfusion of Skin and Muscle Microcirculation? A Pilot Feasibility Study on Healthy Subjects. J. Altern. Complement. Med..

[7] Sadri S., Kaka G., Manshadi F.D., Yazdani A., Gholami H., Shabanloei R., Ahmadi K. (2022). Effects of Transfer Energy Capacitive and Resistion On Musculoskeletal Pain: A Systematic Review and Meta-Analysis. Galen. Med. J..

[8] Kudich E., Miha V., Kozinc Ž. (2025). Effectiveness of TECAR Therapy for Managing Pain in Sports-Related Musculoskeletal Pathologies: A Systematic Review with Meta-analysis. SN Compr. Clin. Med..

[9] López-Garrido A., González-Gutiérrez M.D., Ibáñez-Vera A.J. (2023). Efectividad de la diatermia por radiofrecuencia en el tratamiento de las patologías de rodilla. Una revisión sistemática. Fisioterapia.

[10] Ribeiro S., Henriques B., Cardoso R. (2018). The Effectiveness of Tecar Therapy in Musculoskeletal Disorders. Int. J. Public Health Health Syst..

[11] Violan E.E., Lalisan E.J., Tablante T.M., Bascon C., Te R.L. (2023). Effectiveness of TECAR Therapy and Therapeutic Exercise in the Treatment of Musculoskeletal Conditions: A Review Article. Int. J. Innov. Sci. Res. Technol..

[12] García-Rueda L., Cabanas-Valdés R., Salgueiro C., Pérez-Bellmunt A., Rodríguez-Sanz J., López-de-Celis C. (2025). Immediate effects of TECAR therapy on lower limb to decrease hypertonia in chronic stroke survivors: A randomized controlled trial. Disabil. Rehabil..

[13] Fontes A.R., Martins A.S.M., Costa B.S.P.D., Noites A., Marques L. (2022). Comparison of the effects of shock waves versus radiofrequency on abdominal lipolysis: A randomized clinical trial. J. Cosmet. Dermatol..

[14] Beltrame R., Ronconi G., Ferrara P.E., Salgovic L., Vercelli S., Solaro C., Ferriero G. (2020). Capacitive and resistive electric transfer therapy in rehabilitation: A systematic review. Int. J. Rehabil. Res..

[15] Szabo D.A., Neagu N., Teodorescu S., Predescu C., Sopa I.S., Panait L. (2022). Therapy Associated with High-Intensity Laser Therapy (Hilt) and Manual Therapy in the Treatment of Muscle Disorders. J. Clin. Med..

[16] Page M.J., McKenzie J.E., Bossuyt P.M., Boutron I., Hoffmann T.C., Mulrow C.D., Shamseer L., Tetzlaff J.M., Akl E.A., Brennan S.E. (2021). The PRISMA 2020 statement: An updated guideline for reporting systematic reviews. BMJ.

[17] Stone P.W. (2002). Popping the (PICO) question in research and evidence-based practice. Appl. Nurs. Res..

[18] Ouzzani M., Hammady H., Fedorowicz Z., Elmagarmid A. (2016). Rayyan—A web and mobile app for systematic reviews. Syst. Rev..

[19] Maher C.G., Sherrington C., Herbert R.D., Moseley A.M., Elkins M. (2003). Reliability of the PEDro Scale for Rating Quality of Randomized Controlled Trials. Phys. Ther..

[20] Higgins J.P., Altman D.G., Gøtzsche P.C., Jüni P., Moher D., Oxman A.D., Savovic J., Schulz K.F., Weeks L., Sterne J.A. (2011). The Cochrane Collaboration’s tool for assessing risk of bias in randomised trials. BMJ..

[21] Higgins J.P.T., Green S. (2019). Cochrane Handbook for Systematic Reviews of Interventions.

[22] Nazari S., Sohani S.M., Sarrafzadeh J., Angoorani H., Tabatabaei A. (2025). The effects of TECAR therapy on pain, range of motion, strength and subscale of HAGOS questionnaire in athletes with chronic adductor related groin pain. BMC Musculoskelet. Disord..

[23] Jang Y., Je L.G., Lee S., Na D., Shin H., Choi J.B., Koh J.C. (2023). Efficacy of Transcutaneous 4.4 MHz Radiofrequency Diathermy versus Therapeutic Ultrasound for Pain Relief and Functional Recovery in Patients with Knee Osteoarthritis. J. Clin. Med..

[24] Karas L. (2024). Book of Abstracts: XVI International Days of Rehabilitation.

[25] Uzun Ö., Sezgin Özcan D., Bölük Şenlikçi H., Atalay Z., Ünal R., Dalyan M. (2025). Clinical effects of TECAR therapy in the conservative management of Stage 2 lipedema in females: A randomized controlled trial. Turk. J. Phys. Med. Rehabil..

[26] Cau N., Cimolin V., Aspesi V., Galli M., Postiglione F., Todisco A., Tacchini E., Darno D., Capodaglio P. (2019). Preliminary evidence of effectiveness of TECAR in lymphedema. Lymphology.

[27] Davari A., Mansour Sohani S., Sarrafzadeh J., Nikjooy A. (2021). Evaluation of the Effects of Tecar Therapy on Acute Symptoms of Athletes Following Lateral Ankle Ligament Sprain. Funct. Disabil. J..

[28] Kelli A., Apostolou T., Iakovidis P., Koutras G., Kellis E. (2025). Acute effects of medium-frequency electrical energy transfer (TECAR) and transcutaneous electrical nerve stimulation (TENS) on pain and flexibility in athletes with an acute hamstring injury. Sports Med. Health Sci..

[29] Albornoz-Cabello M., Ibáñez-Vera A.J., Barrios-Quinta C.J., Espejo-Antúnez L., Lara-Palomo I.C., de Los Ángeles Cardero-Durán M. (2024). Non-Invasive Radiofrequency Diathermy Neuromodulation Added to Supervised Therapeutic Exercise in Patellofemoral Pain Syndrome. Biomedicines.

[30] Tezen Ö., Bilir E.E., Uzun Ö., Yaniktaş D., Şentürk B., Yaşar E. (2024). Evaluation of the efficacy of transfer energy capacitive and resistive therapy in patients with knee osteoarthritis. Turk. J. Med. Sci..

[31] Albornoz-Cabello M., Ibáñez-Vera A.J., Barrios-Quinta C.J., Lara-Palomo I.C., Cardero-Durán M.L.Á., Espejo-Antúnez L. (2023). Effects of Radiofrequency Diathermy Plus Therapeutic Exercises on Pain and Functionality of Patients with Patellofemoral Pain Syndrome. J. Clin. Med..

[32] Albornoz-Cabello M., Ibáñez-Vera A.J., Aguilar-Ferrándiz M.E., Espejo-Antúnez L. (2020). Monopolar dielectric diathermy by emission of radiofrequency in Patellofemoral pain. Electromagn. Biol. Med..

[33] Coccetta C.A., Sale P., Ferrara P.E., Specchia A., Maccauro G., Ferriero G., Ronconi G. (2019). Effects of capacitive and resistive electric transfer therapy in patients with knee osteoarthritis: A randomized controlled trial. Int. J. Rehabil. Res..

[34] Kumaran B., Watson T. (2019). Treatment using 448kHz capacitive resistive monopolar radiofrequency improves pain and function in patients with osteoarthritis of the knee joint. Physiotherapy.

[35] García-Marín M., Rodríguez-Almagro D., Castellote-Caballero Y., Achalandabaso-Ochoa A., Lomas-Vega R., Ibáñez-Vera A.J. (2021). Efficacy of non-invasive radiofrequency-based diathermy in the postoperative phase of knee arthroplasty. J. Clin. Med..

[36] García-Rueda L., Cabanas-Valdés R., Salgueiro C., Rodríguez-Sanz J., Pérez-Bellmunt A., López-de-Celis C. (2023). Immediate Effects of TECAR Therapy on Gastrocnemius and Quadriceps Muscles with Spastic Hypertonia in Chronic Stroke Survivors. Biomedicines.

[37] Javan Amoli M., Khademi-Kalantari K., Niajalili M., Daryabor A., Sadat Naimi S. (2024). Comparison of the effect of separate and simultaneous application of Tecar therapy and low-level laser therapy on type 2 diabetic patients. Turk. J. Phys. Med. Rehabil..

[38] Niajalili M., Mohsen Roostayi M., Daryabor A., Naimi S.S., Amoli M.J. (2023). The effect of Tecar therapy on neurological disorders and nerve conduction velocity of lower limbs in type 2 diabetic patients. Turk. J. Phys. Med. Rehabil..

[39] Niajalili M., Sedaghat M., Reazasoltani A., Baghban A.A., Naimi S.S. (2020). Effect of Capacitive Tecar Therapy on Foot Pain and Tactile Sensation in Patients with Type 2 Diabetes. J. Rehabil..

[40] Lupowitz L.G., Ramus L., Delacour F., Johnson K. (2025). TECAR Therapy: A Clinical Commentary on its Evolution, Application, and Future in Rehabilitation. Int. J. Sports Phys. Ther..

[41] He P., Fu W., Shao H., Zhang M., Xie Z., Xiao J., Li L., Liu Y., Cheng Y., Wang Q. (2023). The effect of therapeutic physical modalities on pain, function, and quality of life in patients with myofascial pain syndrome: A systematic review. BMC Musculoskelet. Disord..

[42] Sorrentino M., Ferrari D., Elena Z.I. (2022). Effectiveness of a Long-Term Tecar Therapy Treatment on Knee Pain. Syst. Rev. Pharm..

[43] Farì G., de Sire A., Fallea C., Albano M., Grossi G., Bettoni E., Di Paolo S., Agostini F., Bernetti A., Puntillo F. (2022). Efficacy of Radiofrequency as Therapy and Diagnostic Support in the Management of Musculoskeletal Pain. Diagnostics.

[44] Bhatia A., Hoydonckx Y., Peng P., Cohen S.P. (2018). Radiofrequency Procedures to Relieve Chronic Hip Pain: An Evidence-Based Narrative Review. Reg. Anesth. Pain Med..

[45] Jitsinthunun T., Li C., Ng T.K., Zinboonyahgoon N. (2025). Narrative Review Pulsed Radiofrequency Treatment: Evidence for and Applications in Chronic Pain. Pain Physician.

[46] Wu W., Shi Y. (2016). Systematic Review Treatment of Neuropathic Pain Using Pulsed Radiofrequency: A Meta-analysis. Pain Physician.

[47] Van Boxem K., Huntoon M., Van Zundert J., Patijn J., van Kleef M., Joosten E.A. (2014). Pulsed radiofrequency: A review of the basic science as applied to the pathophysiology of radicular pain. Reg. Anesth. Pain Med..

[48] Watson T., Nussbaum E. (2020). Electrophysical Agents: Evidence-Based Practice.

[49] Rodríguez-Sanz J., Pérez-Bellmunt A., López-de-Celis C., Lucha-López O.M., González-Rueda V., Tricás-Moreno J.M., Simon M., Hidalgo-García C. (2020). Thermal and non-thermal effects of capacitive-resistive electric transfer application on different structures of the knee: A cadaveric study. Sci. Rep..

