# Supplementary material for: Non-Invasive Radiofrequency Therapy for Musculoskeletal, Neurological, and Vascular Conditions of the Lower Limb: A Systematic Review and Meta-Analysis
Source: J Clin Med. 2026 Mar 22;15(6):2428. doi: 10.3390/jcm15062428 (PMC13027387; doi:10.3390/jcm15062428)
Supplement: Supplementary file 1 [file jcm-15-02428-s001.zip › Supplementary S2.pdf]

Search strategy

PubMed

(("Radiofrequency Therapy"[Mesh]

OR "radiofrequency"[tiab]

OR "radio-frequency"[tiab]

OR "RF therapy"[tiab]

OR "diathermy"[tiab]

OR "tecar"[tiab]

OR "tecar therapy"[tiab]

OR "transfer of energy capacitive and resistive"[tiab])

AND

("Lower Extremity"[Mesh]

OR "lower limb"[tiab]

OR "lower extremity"[tiab]

OR "leg"[tiab]

OR "knee"[tiab]

OR "ankle"[tiab]

OR "hip"[tiab]

OR "foot"[tiab])

AND

("musculoskeletal"[tiab]

OR "pain"[tiab]

OR "rehabilitation"[tiab]

OR "physiotherapy"[tiab]

OR "physical therapy"[tiab]))

NOT

("catheter ablation"[tiab]

OR "nerve ablation"[tiab]

OR "tumor"[tiab]

OR "neoplasm"[tiab]

OR "cancer"[tiab]

OR "surgery"[tiab]

OR "surgical"[tiab]

OR "neurotomy"[tiab])

503 artículos poniendo clinical trials y Randomized Controlled Trial 93

...

SCOPUS

(TITLE-ABS-KEY("radiofrequency therapy"

OR "radiofrequency"

OR "radio-frequency"

OR "RF therapy"

OR "diathermy"

OR "tecar"

OR "tecar therapy"

OR "transfer of energy capacitive and resistive")

AND

TITLE-ABS-KEY("lower extremity"

OR "lower limb"

OR "leg"

OR "knee"

OR "ankle"

OR "hip"

OR "foot")

AND

TITLE-ABS-KEY("musculoskeletal"

OR "pain"

OR "rehabilitation"

OR "physiotherapy"

OR "physical therapy"))

AND NOT

TITLE-ABS-KEY("catheter ablation"

OR ablation

OR "nerve ablation"

OR "tumor"

OR "neoplasm"

OR "cancer"

OR "surgery"

OR "surgical"

OR "neurotomy")

524 documents quitando review, letter, conference paper, book chapter y limitando a artículos 373 y luego cuando lo espefiqué en áreas de salud se obtuvo 349

In Scopus, search results were limited to the subject areas “Medicine” and “Health Professions” to ensure the inclusion of clinically relevant studies. In addition, document types such as reviews, letters, conference papers, and book chapters were excluded. Only original research articles were considered. After applying these filters, 373 records were identified.

WOS
("radiofrequency therapy" OR "radiofrequency" OR "radio-frequency" OR "RF therapy" OR "diathermy" OR "tecar" OR "tecar therapy" OR "transfer of energy capacitive and resistive") AND ("lower extremity" OR "lower limb" OR "leg" OR "knee" OR "ankle" OR "hip" OR "foot") AND ("musculoskeletal" OR "pain" OR "rehabilitation" OR "physiotherapy" OR "physical therapy") NOT ("catheter ablation" OR ablation OR "nerve ablation" OR "tumor" OR "neoplasm" OR "cancer" OR "surgery" OR "surgical" OR "neurotomy")

456 artículos quitando case report, review article, me quedaron 276 artículos y cuando le especifque las áreas 192 documentos

In Web of Science, search results were limited to the subject areas “Medicine” and “Health Professions” to ensure clinical relevance. Document types such as review articles, clinical trials, case reports, meeting abstracts, dissertations/theses, letters, patents, book chapters, early access, corrections, and retracted publications were excluded. Only original research articles were included, resulting in records.

…

Scielo

("radiofrequency" OR "radio-frequency" OR "RF therapy" OR "diathermy" OR "tecar" OR "tecar therapy" OR "transfer of energy capacitive and resistive") AND ("lower limb" OR "lower extremity" OR "leg" OR "knee" OR "ankle" OR "hip" OR "foot") AND ("musculoskeletal" OR "pain" OR "rehabilitation" OR "physiotherapy" OR "physical therapy")

Y salieron 15 artículos

En rayyan export 14 ris

COCHRANE

("radiofrequency therapy" OR "radiofrequency" OR "radio-frequency" OR "RF therapy" OR "diathermy" OR "tecar" OR "tecar therapy" OR "transfer of energy capacitive and resistive") AND ("lower limb" OR "lower extremity" OR "leg" OR "knee" OR "ankle" OR "hip" OR "foot") AND ("musculoskeletal" OR "pain" OR "rehabilitation" OR "physiotherapy" OR "physical therapy") NOT ("catheter ablation" OR ablation OR "nerve ablation" OR "tumor" OR "neoplasm" OR "cancer" OR "surgery" OR "surgical" OR "neurotomy")

SALIERON 634 ENSAYOS

ESO ES CIATATION EPOR 18
